# Toxicity of Water-Soluble D-g-PNIPAM Polymers in a Complex with Chemotherapy Drugs and Mechanism of Their Action In Vitro

**DOI:** 10.3390/ijms25053069

**Published:** 2024-03-06

**Authors:** Svitlana Prylutska, Anna Grebinyk, Stanislav Ponomarenko, Defne Gövem, Vasyl Chumachenko, Nataliya Kutsevol, Mykola Petrovsky, Uwe Ritter, Marcus Frohme, Jacek Piosik, Yuriy Prylutskyy

**Affiliations:** 1 Department of Plants Physiology, Biochemistry and Bioenergetics, National University of Life and Environmental Sciences of Ukraine, 03041 Kyiv, Ukraine; psvit_1977@ukr.net; 2 Deutsches Elektronen-Synchrotron DESY, Platanenallee 6, 15738 Zeuthen, Germany; anna.grebinyk@desy.de; 3 Division Molecular Biotechnology and Functional Genomics, Technical University of Applied Sciences Wildau, 15745 Wildau, Germany; defne.govem@hotmail.com (D.G.); mfrohme@th-wildau.de (M.F.); 4 Department of Biophysics and Neurobiology, Department of Chemistry, Taras Shevchenko National University of Kyiv, 01601 Kyiv, Ukraine; stasponomarenko@ukr.net (S.P.); vasyl.chumachenko@knu.ua (V.C.); nataliya.kutsevol@knu.ua (N.K.); mykola.petrovsky@ukr.net (M.P.); prylut@ukr.net (Y.P.); 5 Institute of Chemistry and Biotechnology, Technical University of Ilmenau, 98693 Ilmenau, Germany; uwe.ritter@tu-ilmenau.de; 6 Intercollegiate Faculty of Biotechnology, University of Gdańsk and Medical University of Gdańsk, 80-307 Gdańsk, Poland

**Keywords:** star-like copolymer, drug, Lewis lung carcinoma, cytotoxicity

## Abstract

The application of a biocompatible polymer nanocarrier can provide target delivery to tumor tissues, improved pharmacokinetics, controlled drug release, etc. Therefore, the proposed strategy was to use the water-soluble star-like copolymers with a Dextran core and Poly(N-isopropylacrylamide) grafts (D-g-PNIPAM) for conjugation with the widely used chemotherapy drugs in oncology–Cisplatin (Cis-Pt) and Doxorubicin (Dox). The molecular characteristics of the copolymer were received using size-exclusion chromatography. The physicochemical characterization of the D-g-PNIPAM-Cis-Pt (or Dox) nanosystem was conducted using dynamic light scattering and FTIR spectroscopy. Using traditional biochemical methods, a comparative analysis of the enhancement of the cytotoxic effect of free Cis-Pt and Dox in combination with D-g-PNIPAM copolymers was performed in cancer cells of the Lewis lung carcinoma line, which are both sensitive and resistant to Dox; in addition, the mechanism of their action in vitro was evaluated.

## 1. Introduction

The conjugation of a drug with a biocompatible polymer that acts as a nanocarrier not only improves its water solubility but also changes the pharmacokinetics of the drug, even at the subcellular level [1,2,3,4]. Therefore, the development of new polymer nanocarriers with a predictable structure and conformation (defined by the architecture in solution), higher homogeneity, greater loading capacity, and increased multifunctionality is an important direction of research in nanomedicine. Currently, the research is focused on the creation of new nonlinear architectures of copolymers [5,6], particularly star-shaped [7], brush-like [8], and hyperbranched structures such as dendrimers [9]. These structures have unique physical and chemical properties compared to linear polymers, making them attractive for use in drug delivery systems [5,10,11].

A high degree of ramification of the polymer is provided by a large number of functional groups, which allow the encapsulation and delivery of drugs with greater efficiency and the targeting of specific tissues or cells in the body [5,11]. Compared to linear analogues, branched polymers have a three-dimensional globular structure and greater mechanical stability, which allows them to withstand the harsh conditions of the body’s environment, lower viscosity, and hydrodynamic size [12,13,14]. These properties contribute to their improved pharmacokinetics, their penetration into cells, their overcoming of biological barriers, etc. [15]. The literature reports the importance of the ratio of hydrophilic/hydrophobic components in such polymeric nanocarriers [16]. Macromolecular architecture is assumed to be a key parameter for tuning the behavior and properties of polymeric systems; thus, it should be taken into account when developing new materials for potential biological applications, particularly those for drug delivery systems [17]. 

Thus, we focused our efforts on the creation of novel nanosystems based on traditional chemotherapeutical drugs such as Doxorubicin (Dox) and Cisplatin (Cis-Pt), encapsulated into star-like Dextran-graft-poly-N-isopropylacrylamide (D-g-PNIPAM) copolymers with various internal macromolecular structures; we also focused on their in vitro testing for potential application in anticancer therapy. 

## 2. Results and Discussion

### 2.1. Physicochemical Characterization of Samples

The peculiarities of the molecular structure of the D-g-PNIPAM copolymers were discussed in detail in [18]. The molecular parameters of the synthesized new D-g-PNIPAM copolymers, determined by the SEC method, are presented in Table 1.

It was reported that Dextran keeps the compact coil conformation in D-g-PNIPAM graft copolymers due to the intermolecular cross-linking caused by Ce(IV) ions [18]. Analysis of the molecular parameters of the synthesized copolymers confirmed that they are star-like and have various grafting efficiencies. It was shown that a decrease in the distance between grafts leads to a more rigid molecular structure, which affects the limited conformational changes and tuning of the hydrophobicity [18,19]. In our opinion, this factor will contribute to the development of pharmaceutical nanomaterials for biomedical applications. 

**Figure 1:**
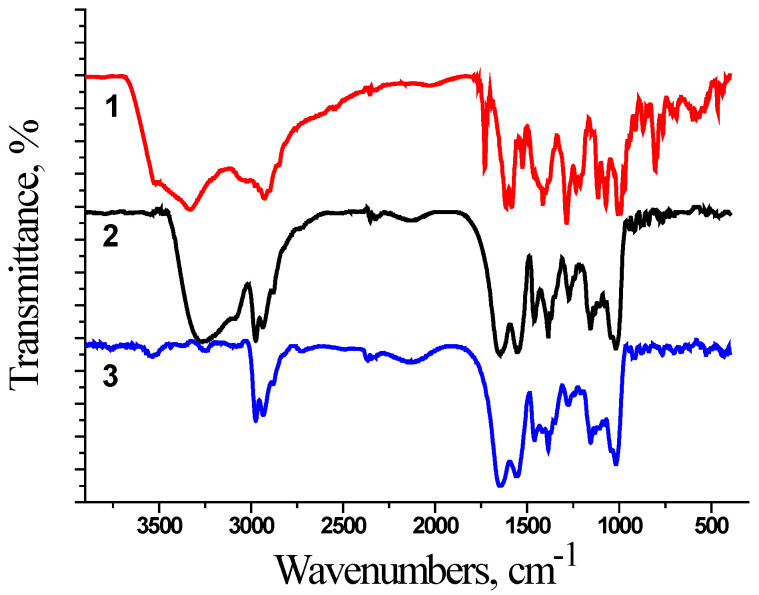
FTIR spectra for: 1—Dox (40 μg/mL); 2—D70-g-PNIPAM (500 μg/mL); and 3—D70-g-PNIPAM-Dox (500 + 40 μg/mL) system.

**Figure 2:**
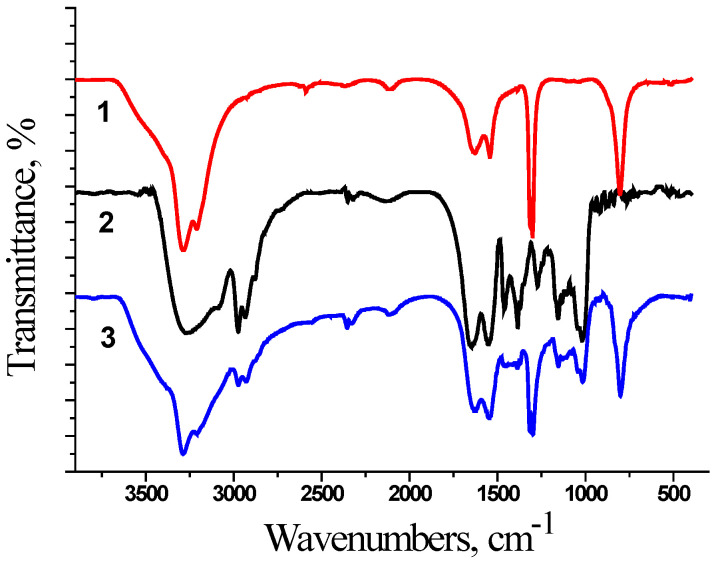
FTIR spectra for 1—Cis-Pt (250 μg/mL); 2—D70-g-PNIPAM (500 μg/mL); and 3—D70-g-PNIPAM-Cis-Pt (500 + 250 μg/mL) system.

At the first stage, the interaction in the polymer–chemotherapeutic drug system was studied by FTIR spectroscopy (Figure 1 and Figure 2). The encapsulation of chemotherapeutic drugs in the polymer nanocarrier was confirmed by the change in the characteristic bands of the polymer, namely in the region of Amide 1 (1650–1660 cm^−1^, C=O) and Amide 2 (1615 cm^−1^, N-H). The FTIR spectra were equal for the synthesized polymer nanocarriers because we used films for the FTIR measurements. In Figure 1 and Figure 2, the spectra for the samples based on D70-g-PNIPAM are shown. 

The FTIR spectra for all the studied systems show a broad band at 3500 cm^−1^ since the measurements were made using films that were poured out of aqueous solution. As the copolymer is hydrophilic, it retains up to 10% of the water even in the dried form. This may be the reason why the bands of the copolymer or composite in the region of 3500–2900 cm^−1^ have a slight difference since there is an overlap of a wide band of the vibrations of the OH groups of water.

For the D70-g-PNIPAM-Dox system (Figure 1), the disappearance of the N-H group of the copolymer (3310 cm^−1^) and the absorption band of the C=O group of Dox (1730 cm^−1^) is observed. This indicates the formation of a complex which mainly consists of amino groups.

The FTIR spectrum of the D70-g-PNIPAM-Cis-Pt system is shown in Figure 2. The spectral changes are not pronounced, but a change in the intensity of the Amide 1 and Amide 2 bands, as well as the C-H bond vibration bands (2980, 2920, 1460, 1380, 1145, and 1030 cm^−1^), is observed.

Thus, the spectral changes for the D70-g-PNIPAM-Dox and D70-g-PNIPAM-Cis-Pt systems in comparison with their components indicate the formation of complexes.

**Figure 3:**
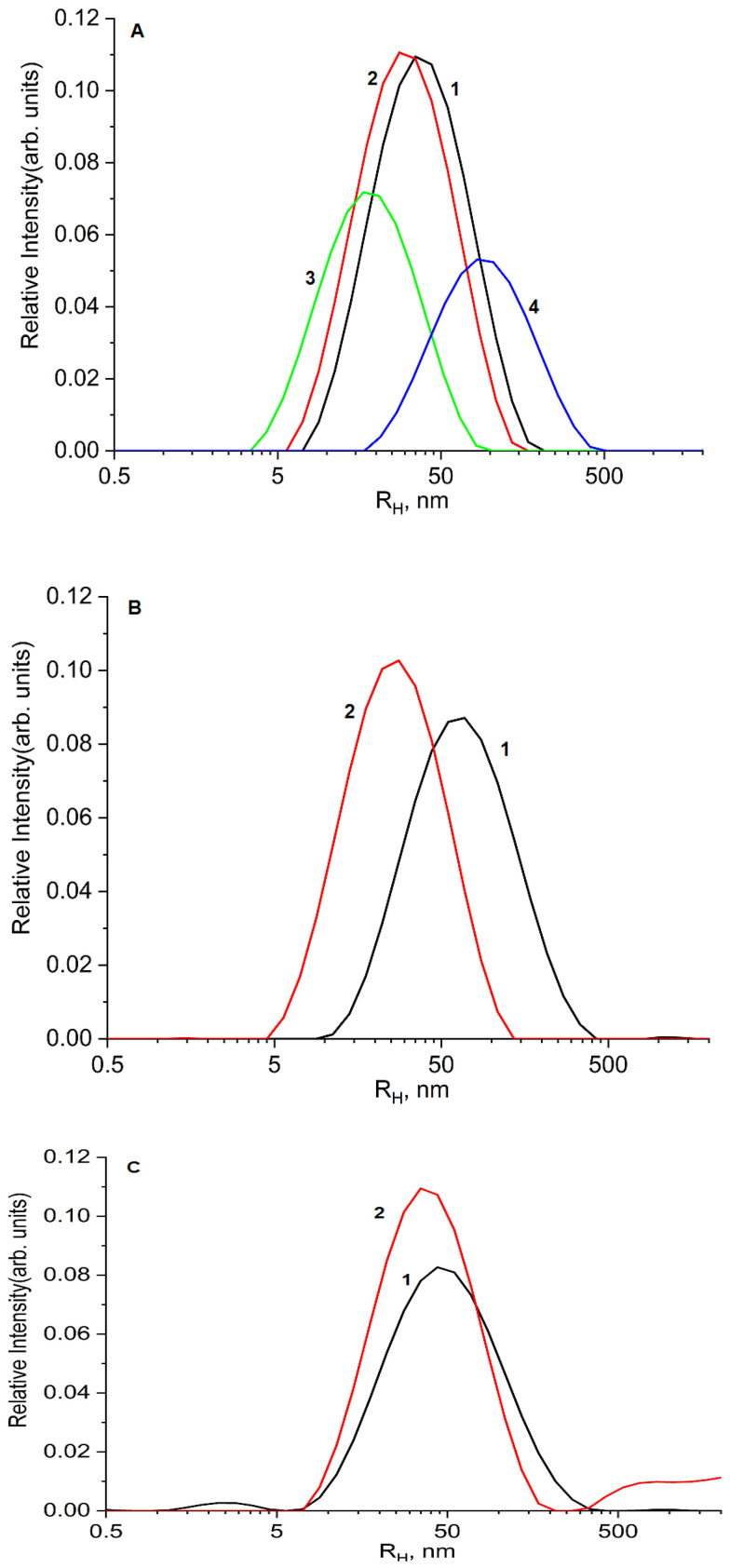
Hydrodynamic radii distribution for individual D70-g-PNIPAM (500 μg/mL) (1), D6-g-PNIPAM (500 μg/mL) (2), Dox (40 μg/mL) (3), and Cis-Pt (250 μg/mL) (4) (**A**) as well as nanosystems D70-g-PNIPAM-Dox (500 + 40 μg/mL) (1), D6-g-PNIPAM-Dox (500 + 40 μg/mL) (2) (**B**) and D70-g-PNIPAM-Cis-Pt (500 + 250 μg/mL) (1), and D6-g-PNIPAM-Cis-Pt (500 + 250 μg/mL) (2) (**C**).

In the second stage, a DLS method was used to study the interaction between the D-g-PNIPAM and the drug molecules as well as the estimation of the particle sizes and their aggregation process (Figure 3). The free aqueous polymers had peaks of intensity at the hydrodynamic radii of 40 nm and 30 nm for D70-g-PNIPAM and D6-g-PNIPAM, respectively, and these values are defined by corresponding molecular weights of the polymers (Figure 3A). The limited solubility of the Dox and Cis-Pt was also revealed in the particle size distribution: R_Hmax_ = 21 and R_Hmax_ = 82 nm for Dox and Cis-Pt, respectively (Figure 3A). It is worth noting that peak intensity is not directly proportional to the concentrations of the components.

The D70-g-PNIPAM-Dox and D6-g-PNIPAM-Dox complexes had peaks of intensity at the hydrodynamic radii of 67 nm and 27 nm, respectively (Figure 3B). The increase in the particle size for the case of D70-g-PNIPAM-Dox was likely caused by the hydrophobic interactions between the PNIPAM chains and Dox, which caused the partial aggregation of the complex. Conversely, the R_Hmax_ of D6-g-PNIPAM-Dox decreased; this was probably also due to the hydrophobic interaction, but the decrease in hydrodynamic radius was due to the compression of this macromolecule without aggregation. Cis-Pt did not have a significant influence on the R_Hmax_ values of the particle size distributions of D70-g-PNIPAM-Cis-Pt and D6-g-PNIPAM-Cis-Pt compared to the bare polymers (Figure 3C). 

The difference in the behavior of the complexes of drugs with the D70-g-PNIPAM and D6-g-PNIPAM nanocarriers is based on the more rigid molecular structure of D6-g-PNIPAM, leading to the limitation of the conformational changes in the grafts and the reduction in the various hydrophobicity levels of the two polymer samples.

### 2.2. Viability of LLC Cells under the Action of Water-Soluble Branched D-g-PNIPAM Polymers in Combination with Chemotherapeutic Agents 

The study of the biological effects of the investigated water-soluble branched D-g-PNIPAM polymers (D6 and D70 modifications), both alone and in combination with chemotherapeutic agents (Dox and Cis-Pt), began with the assessment of their cytotoxic effects, namely their impact on the viability of sensitive and Dox-resistant LLC cells 24 and 48 h after incubation. Cell viability without additives was taken as 100% (control). 

**Figure 4:**
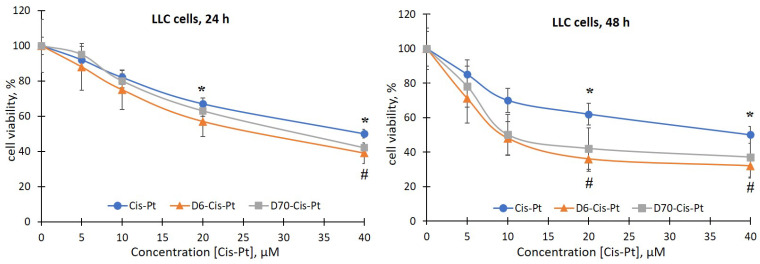
Viability of LLC cells after 24 and 48 h of incubation with free Cis-Pt or in the composition of water-soluble branched D6- and D70-g-PNIPAM-Cis-Pt polymers in an equivalent concentration of Cis-Pt (M ± m, n = 5). * *p* < 0.05 compared to the control; ^#^ *p* < 0.05 compared to the action of free Cis-Pt.

**Figure 5:**
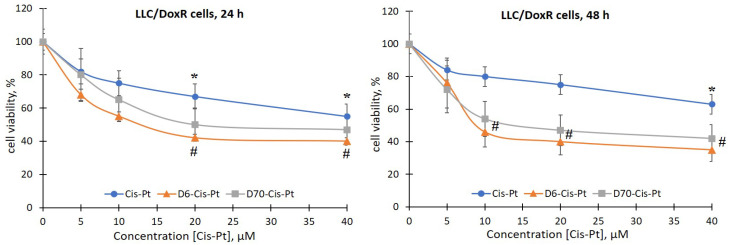
Viability of LLC/DoxR cells after 24 and 48 h of incubation with free Cis-Pt or in the composition of water-soluble branched D6- and D70-g-PNIPAM-Cis-Pt polymers at equivalent concentrations of Cis-Pt (M ± m, n = 5). * *p* < 0.05 compared to the control; ^#^ *p* < 0.05 compared to the effect of free Cis-Pt.

**Figure 6:**
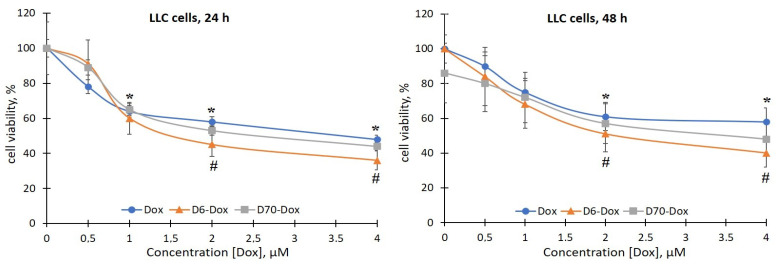
Viability of LLC cells after 24 and 48 h of incubation with free Dox or in the composition of water-soluble branched D6- and D70-g-PNIPAM-Dox polymers at equivalent concentrations of Dox (M ± m, n = 5). * *p* < 0.05 compared to the control; ^#^ *p* < 0.05 compared to the effect of free Dox.

**Figure 7:**
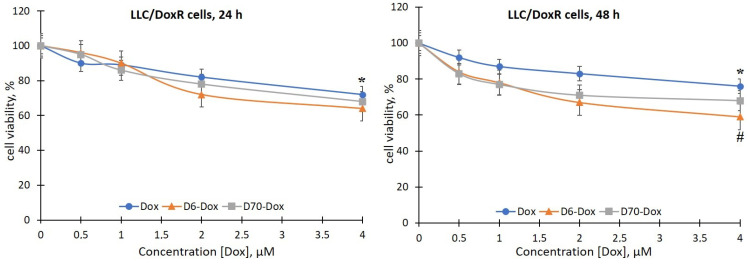
Viability of LLC/DoxR cells after 24 and 48 h of incubation with free Dox or in the composition of water-soluble branched D6- and D70-g-PNIPAM-Dox polymers at equivalent concentrations of Dox (M ± m, n = 5). * *p* < 0.05 compared to the control; ^#^ *p* < 0.05 compared to the effect of free Dox.

There was no effect of the D6- and D70-g-PNIPAM polymers at a concentration range of 5–20 μg/mL on the viability of LLC cells during the studied incubation periods. At the same time, under the action of the Dox and Cis-Pt chemotherapeutic agents, a time- and dose-dependent toxic effect on both the sensitivity and the resistance to Dox LLC cells was detected (Figure 4, Figure 5, Figure 6 and Figure 7).

The viability of LLC cells 24 h after exposure to 20 and 40 μM Cis-Pt decreased by 33% and 50%, respectively, compared to the control (Figure 4). After 48 h, the time-dependent cytotoxic effect of Cis-Pt did not increase, i.e., the viability of the LLC cells was at the level of 24 h of incubation (Figure 4). 

The viability of LLC/DoxR cells under the influence of 20 and 40 μM Cis-Pt decreased by 33% and 45%, respectively, after 24 h of incubation compared to the control and by 25% and 33%, respectively, after 48 h (Figure 5).

Thus, Cis-Pt in the concentration range of 20–40 μM had a toxic effect on both the sensitive and the resistant LLC cells.

The toxicity of Dox for the LLC cells (sensitive and resistant lines) compared to Cis-Pt was 10 times lower, as evidenced by the range of effective concentrations; in particular, the range for Cis-Pt was 10–40 μM, and for Dox—1–4 μM (Figure 6 and Figure 7). 

The viability of the sensitive LLC cells 24 h after exposure to Dox at concentrations of 0.5, 1, 2, and 4 μM decreased by 22%, 36%, 42%, and 52%, respectively, compared to the control (Figure 6). After 48 h, the negative effects of Dox were observed after incubation of the LLC cells in the presence of 1 μM (the number of viable cells decreased by 25%), 2 μM (by 39%), and 4 μM (by 42%) of the chemotherapeutic agent. According to the results obtained, the Dox-resistant LLC cell line was resistant to the toxic effect of Dox: the viability of LLC/DoxR cells after exposure to 4 μM Dox after 24 and 48 h of incubation decreased by only 28% and 24%, respectively, compared to the control (Figure 7).

The results of the toxic effect of the chemotherapeutic agents on the LLC cells were also confirmed by the IC_50_ value (Table 2). In particular, the IC_50_ values 24 and 48 h after Cis-Pt exposure for the sensitive LLC cells were 45.86 μM and 40.30 μM, respectively, and for the LLC/DoxR cells, they were 52.82 μM and 78.98 μM, respectively. The IC_50_ values at 24 and 48 h after Dox exposure for the sensitive LLC cells were 2.89 μM and 4.33 μM, respectively, and for the LLC/DoxR cells, they were 10.38 μM and 11.90 μM, respectively. 

The data obtained indicate the resistance of LLC cells to the toxic effects of chemotherapeutic agents, and the values of the IC_50_ index under their action are consistent with the literature data. Thus, the IC_50_ value after Cis-Pt exposure after 24 h for the LLC cells was ≥50 μM [20], and for the A549 lung cancer cells, it was 55 μM [21] and 64 μM [22], respectively. 

The IC_50_ value of Dox after 24 h for the A549 cells was 0.13 μM [23] and 2 μM [24]. The IC_50_ value for the A549 cells 48 h after exposure to Dox was 0.6 μM, and after 72 h, it was 0.23 μM [25]. 

Also, we evaluated the effect of the complexes of the water-soluble branched polymers with chemotherapeutic agents on the viability of the sensitive and resistant LLC cells. It has been shown that 24 h after the addition of the polymer (D6- or D70-g-PNIPAM)–chemotherapeutic agent (Cis-Pt or Dox) complexes to the suspension of sensitive LLC cells, a slight increase in the toxic effects of chemotherapeutic agents was observed (Figure 4 and Figure 6). At the same time, after 48 h, the viability of the LLC cells was significantly reduced after exposure to 10, 20, and 40 μM of D6-g-PNIPAM-Cis-Pt at an equivalent concentration of Cis-Pt by 22%, 26%, and 18%, respectively; in the presence of 10 and 20 μM of D70-g-PNIPAM-Cis-Pt at an equivalent Cis-Pt concentration, it was reduced by 20% compared to the Cis-Pt alone, and in the presence of 4 μM D6-g-PNIPAM-Dox, it was reduced by 18% compared to the Dox alone at an equivalent concentration (Figure 4 and Figure 6). 

It is important to note that the sensitivity of LLC/DoxR cells to the action of the investigated complexes of polymers with chemopreparations was higher compared to the sensitive LLC cells, which indicates the enhancement of the cytotoxic effects of chemotherapeutic agents by polymers and the modification of the processes of cell resistance to cytostatics. In particular, the viability of LLC/DoxR cells after exposure to D6-g-PNIPAM-Cis-Pt and D70-g-PNIPAM-Cis-Pt decreased in a time- and concentration-dependent manner: thus, 24 h after incubation of LLC/DoxR cells under exposure to 10 and 20 μM D6-g-PNIPAM-Cis-Pt, this indicator decreased by 20% and 25%, respectively, and after 48 h—by 34% and 35%, respectively, compared to the individual action of Cis-Pt at an equivalent concentration (Figure 5 and Figure 7). After exposure to D70-g-PNIPAM-Cis-Pt, the cytotoxic effect was observed only after 48 h, i.e., the viability of the LLC/DoxR cells decreased by 26%, 28%, and 21% at complex concentrations of 1, 2, and 4 μM, respectively, compared to the separate action of Cis-Pt in an equivalent concentration. Under the influence of 4 μM D6-g-PNIPAM-Dox, the viability of the LLC/DoxR cells was reduced by 17% compared to the separate action of Dox at an equivalent concentration after 48 h (Figure 5 and Figure 7). 

It was found that the toxicity of Cis-Pt in complex with D6- or D70-g-PNIPAM polymers to LLC cells increases at low concentrations of this chemotherapeutic agent. More pronounced toxic effects were observed after exposure to the D6-g-PNIPAM-Cis-Pt complex after 48 h of incubation, as evidenced by a twofold decrease in the viability of the sensitive and resistant LLC cells compared to the individual effect of Cis-Pt at an equivalent concentration (Figure 4 and Figure 5). 

The next step was to investigate the biochemical mechanisms of the cytotoxic action of D-g-PNIPAM polymer complexes with chemotherapeutic agents that can be realized at the cytosolic level. 

### 2.3. ROS Production in LLC Cells under the Influence of Water-Soluble D-g-PNIPAM Polymers in Combination with Chemotherapeutic Agents 

To elucidate the mechanisms of the toxic effect of the studied compounds, which are realized through the induction of oxidative stress, we evaluated the production of ROS in sensitive and resistant LLC cells 18 h after the addition of chemotherapeutic agents, polymers, and their complexes using the DCF-DA fluorescent probe and spectrofluorimetry. ROS production in the cells without additives was taken as 100% (control). 

**Figure 8:**
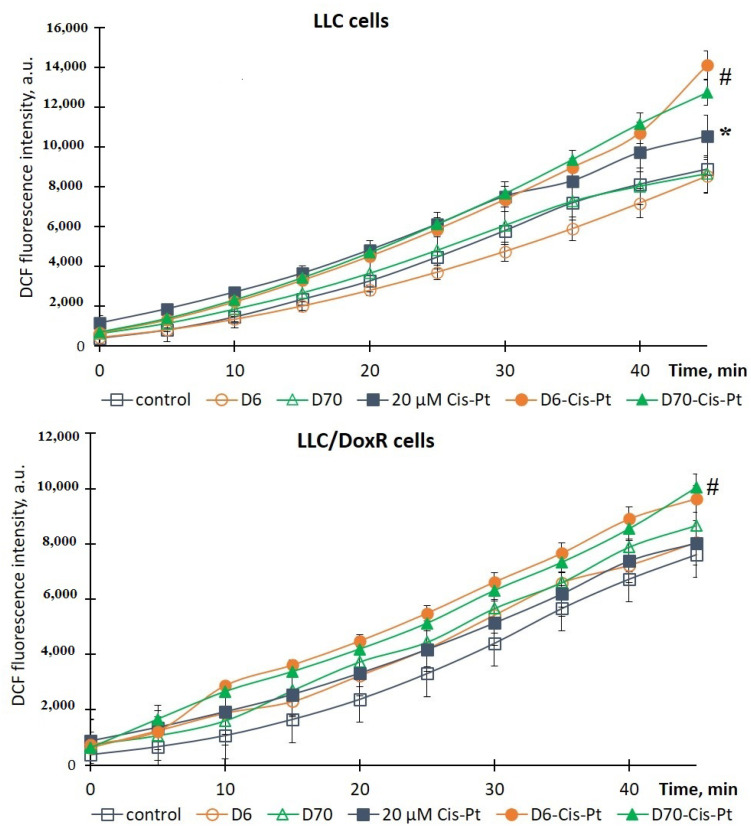
Dynamics of ROS production in LLC and LLC/DoxR cells after 18 h in control, under the action of free Cis-Pt or in the composition of water-soluble D6- or D70-g-PNIPAM-Cis-Pt polymers in an equivalent concentration of Cis-Pt (M ± m, n = 5). * *p* < 0.05 compared to the control; ^#^ *p* < 0.05 compared to the action of free Cis-Pt at an equivalent concentration.

**Figure 9:**
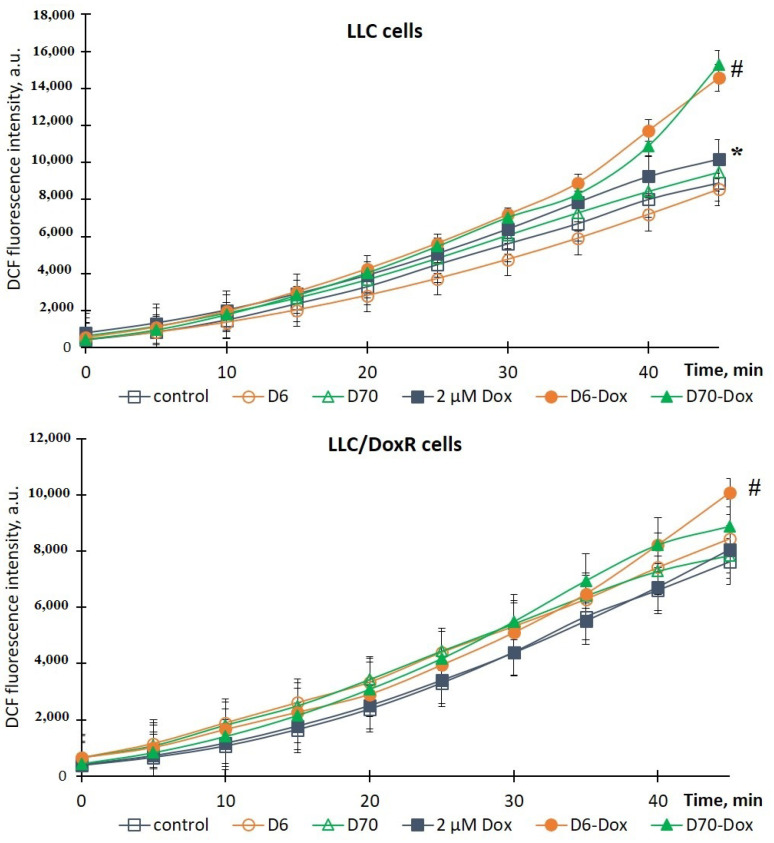
Dynamics of ROS production in LLC and LLC/DoxR cells after 18 h in the control, under the action of free Dox or in the composition of water-soluble D6- or D70-g-PNIPAM-Dox polymers in an equivalent concentration of Dox (M ± m, n = 5). * *p* < 0.05 compared to the control; ^#^ *p* < 0.05 compared to the action of free Dox at an equivalent concentration.

It was shown that the water-soluble D6- or D70-g-PNIPAM polymers did not induce ROS production in the LLC and LLC/DoxR cells after 18 h of incubation (Figure 8 and Figure 9), and the values were close to the control values. 

The chemotherapeutic agents, particularly Cis-Pt and Dox, are known as inducers of oxidative stress, the cytotoxic effect of which is accompanied by excessive ROS production [26,27]. Thus, under the action of 20 μM Cis-Pt and 2 μM Dox, the level of ROS in the LLC cells increased by 19% and 15%, respectively, compared to the control for 45 min (Figure 8 and Figure 9). At the same time, the chemotherapeutic agents did not induce ROS production in the LLC/DoxR cells, indicating the resistant properties of this cell line. 

It is noteworthy that the production of ROS in both cell lines (sensitive LLC and resistant LLC/DoxR) was significantly increased by the action of chemotherapeutic agents with the D6- or D70-g-PNIPAM polymers. In particular, the intracellular level of ROS in the LLC and LLC/DoxR cells increased by 34% and 20%, respectively, after 18 h of exposure to D6-g-PNIPAM-Cis-Pt compared to the free Cis-Pt, and by 21% and 25%, respectively, after exposure to D70-g-PNIPAM-Cis-Pt compared to the free Cis-Pt at an equivalent concentration (Figure 8). 

This indicator increased after the action of D6-g-PNIPAM-Dox in the LLC and LLC/DoxR cells after 18 h by 43% and 25%, respectively, compared to the action of the free Dox, and after the action of D70-g-PNIPAM-Dox–, it increased by 51% and 10%, respectively, compared to the action of the free Dox at an equivalent concentration (Figure 9). 

Therefore, the toxicity of the chemotherapeutic agents (Cis-Pt or Dox) increased upon complexation with the water-soluble D-g-PNIPAM polymers (D6 or D70 modifications), as evidenced by the increased intracellular level of reactive oxygen species (ROS) in both the LLC and the LLC/DoxR cells. 

Both the complexes of D-g-PNIPAM (D6 or D70 modifications) with chemotherapeutic agents (Cis-Pt or Dox) exhibited effective antitumor activity against the LLC cells, while only the complex of D6-g-PNIPAM-Cis-Pt showed such activity against the LLC/DoxR cells. 

### 2.4. ATP Content in LLC Cells under the Action of Water-Soluble D-g-PNIPAM Polymers in A Complex with Chemotherapeutic Agents

Mitochondria play an important role in the induction of apoptosis and are intracellular targets for cytostatics that cause oxidative damage to mitochondria [28,29]. Therefore, we studied the effect of the investigated compounds on the formation of ATP as the basis of the functioning of mitochondria. 

**Figure 10:**
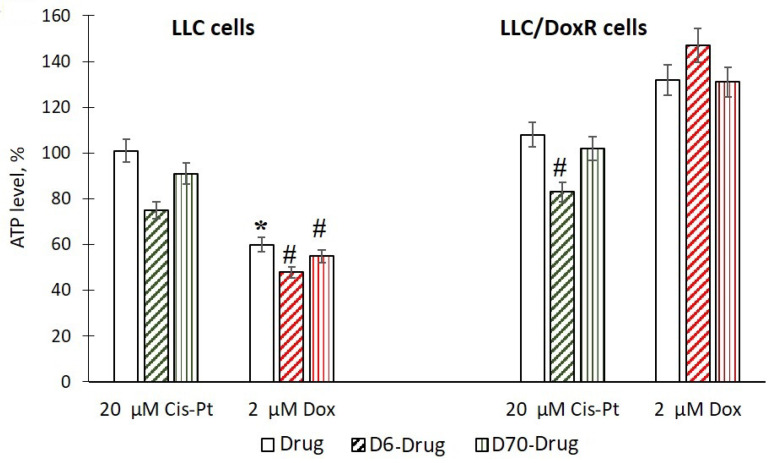
ATP level in LLC and LLC/DoxR cells after 24 h in control, under the action of a free chemotherapeutic agent or in the composition of water-soluble D6 or D70-g-PNIPAM-Cis-Pt polymers and D6 or D70-g-PNIPAM-Dox at equivalent chemotherapeutic agent concentration (M ± m, n = 5). * *p* < 0.05 compared to control; ^#^ *p* < 0.05 compared to the action of a free chemotherapeutic agent at equivalent concentration.

It was shown that the level of ATP decreased in the LLC cells only under the influence of 2 μM Dox, while under other experimental conditions, the studied indicator corresponded to the control values or increased significantly (Figure 10). These results may indicate a violation of the structural integrity and dysfunction of mitochondria [30,31]. 

A significant decrease in ATP levels of 26% and 23% was observed after exposure to D6-g-PNIPAM-Cis-Pt at 24 h in the LLC and LLC/DoxR cells, respectively, compared to exposure to free Cis-Pt at an equivalent concentration, as well as a decrease of 20% after exposure to D6-g-PNIPAM-Dox on LLC cells compared to the action of free Dox at an equivalent concentration (Figure 10). After treatment of the LLC and LLC/DoxR cells with D70-g-PNIPAM-Cis-Pt or D70-g-PNIPAM-Dox, this indicator did not change (Figure 10). 

Therefore, a significant enhancement of the cytotoxic effect of chemotherapeutic agents at the level of mitochondria was observed when complexed with the water-soluble D6-g-PNIPAM polymer. 

### 2.5. Activation of Caspase 3/7 in LLC Cells under the Action of Water-Soluble Polymers D-g-PNIPAM in A Complex with Chemotherapeutic Agents

The next task was to determine the mechanism of the cytotoxic action of the studied compounds by inducing apoptosis. For this purpose, we studied the activity of the terminal protease caspase 3/7, which is activated during caspase-dependent apoptosis [32]. 

**Figure 11:**
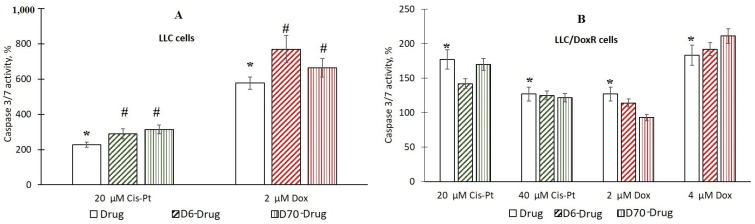
Activation of caspase 3/7 in LLC (**A**) and LLC/DoxR cells (**B**) after 24 h in control, under the action of a free chemotherapeutic agent or in the composition of water-soluble D6- or D70-g-PNIPAM-Cis-Pt polymers and D6- or D70-g-PNIPAM-Dox at equivalent concentrations of chemotherapeutic agents (M ± m, n = 5). * *p* < 0.05 compared to the control; ^#^ *p* < 0.05 compared to the effect of free chemotherapeutic agent at an equivalent concentration.

It was shown that after the action of the chemotherapeutic agents, after 24 h of incubation of the LLC and LLC/DoxR cells, the activity of the enzyme significantly increased, by 2 and 1.7 times under the action of 20 µM Cis-Pt and by 5.7 and 1.3 times under the action of 2 µM Dox, respectively, compared to the control cells (Figure 11).

The authors in [33] disclosed the activation of caspase-3 in HepG2 Huh7 and HeLa tumor cells 36 h after the action of 1 μM Dox. The study in [34] demonstrated the cleavage of caspase-3 in A549 lung cancer cells under the action of 25 μM Cis-Pt. 

We did not detect caspase 3/7 activation 24 h after exposure to the water-soluble D6- or D70-g-PNIPAM polymers in the LLC and LLC/DoxR cells. At the same time, in the LLC cells, after exposure to D6-g-PNIPAM-Cis-Pt and D6-g-PNIPAM-Dox, the caspase 3/7 activity increased by 28% and 34%, respectively, compared to the free chemotherapeutic agents at equivalent concentrations, and after exposure to D70-g-PNIPAM-Cis-Pt and D70-g-PNIPAM-Dox, it increased by 38% and 15%, respectively, compared to the free chemotherapeutic agents at equivalent concentrations (Figure 11A). In the resistant LLC/DoxR cells, no increase in the concentration-dependent caspase activation caused by Cis-Pt and Dox upon complexation with the water-soluble D6- or D70-g-PNIPAM polymers was detected (Figure 11B). 

Thus, a comparative analysis of the antitumor activity of the obtained samples in vitro showed that the cytotoxic action of the complexes of the chemotherapeutic agents (Cis-Pt and Dox) with the water-soluble D-g-PNIPAM polymers (D6 and D70 modifications) is more effective than the individual action of the chemotherapeutic agents; this was confirmed by our preliminary results on their complex formation with water-soluble C_60_ fullerene nanoparticles [35,36].

## 3. Materials and Methods

### 3.1. Copolymer Nanocarrier Synthesis

For the synthesis of star-like graft copolymers with a Dextran core and a grafted Poly-N-isopropylacrylamide (PNIPAM) arm, we used Dextrans (Merck, Darmstadt, Germany) with the characteristics given by the manufacturer of M_w_ = 6 × 10^3^ and M_w_ = 70 × 10^3^ (designated as D6 and D70 throughout) and N-isopropylacrylamide (NIPAM) from Merck. Cerium (IV) ammonium nitrate from Merck was an initiator of the radical grafted polymerization. Monomer NIPAM was twice recrystallized from hexane and dried under vacuum for 24 h at room temperature. The number of grafts per Dextran backbone was determined by the molar ratio of the NIPAM monomer to cerium ions, and it was equal to 15. The amount of monomer NIPAM was the same for both grafting processes to D6 and D70. The synthesized samples were marked as D6-g-PNIPAM and D70-g-PNIPAM. 

### 3.2. Water-Soluble D-g-PNIPAM-Dox and D-g-PNIPAM-Cis-Pt Nanocomposites Creation 

The chemotherapeutic drugs Dox and Cis-Pt were obtained from Merck (Darmstadt, Germany). A stock solution of D-g-PNIPAM copolymer (1000 μg/mL) was prepared in distilled water. This copolymer (D6 or D70 modification) and the drug (Dox or Cis-Pt) were mixed to obtain water-soluble D-g-PNIPAM + Dox (500 + 40 μg/mL) or D-g-PNIPAM + Cis-Pt (500 + 250 μg/mL) nanosystems. Thus, the stock concentrations of Dox and Cis-Pt in the D-g-PNIPAM-Dox and D-g-PNIPAM-Cis-Pt nanocomposites were 74 and 830 μM, respectively. 

### 3.3. Size-Exclusion Chromatography

Multi-detection size-exclusion chromatography (SEC) using a specific SEC line enables the determination of the molecular weight distribution and average molecular weights of the branched PNIPAM sample. The SEC line involved a usual HPLC part (Shimadzu, Kyoto, Japan) and Malvern’s triple detection TDA 302 (Viscotek, London, UK), incorporating a refractometer, a viscosimeter, and a two-angle (7 and 90°) light scattering apparatus. The fractionation was carried out through three columns of PLgel Mixed B with a pre-column arranged in series. The eluent was N-methyl-2-pyrrolidone of HPLC grade with 0.1 M LiBr. Measurements were performed at 60 °C using a constant flow rate of 0.5 mL/min. A PNIPAM solution with a concentration of 3.33 g/L was filtered on a 0.45 mm membrane before being injected. A volume of 100 mL of the solution was injected. 

### 3.4. Dynamic Light Scattering

Dynamic light scattering (DLS) measurements were carried out using the Zetasizer Nano ZS90 (Malvern Instruments Ltd., Malvern, UK). The apparatus contains a 4 mW He-Ne laser with a wavelength of 632.8 nm, and the scattered light is detected at an angle of 173° (backscattering) at 25 °C. The regularized singular value decomposition analysis of the averaged correlograms for the studied samples was performed to estimate particle size distribution. 

### 3.5. Fourier-Transform Infrared Spectroscopy

The Fourier-transform infrared spectroscopy (FTIR) spectra were recorded using a Nicolet NEXUS-475 spectrometer (Waltham, MA, USA) in the wavelength range of 4000–400 cm^−1^. Thin films obtained from an aqueous solution of the D-g-PNIPAM copolymer, as well as the copolymer solutions with the addition of Dox or Cis-Pt at 25 °C, were prepared. The thickness of the films was 6–9 µm. 

### 3.6. Tumor Cell Lines and Conditions of Their Cultivation 

The tumor cell lines used in the study were mouse Lewis lung carcinoma (LLC) cells and their Dox-resistant line (LLC/DoxR). The cell lines were obtained from the collection of the Cell Cultures of the R.E. Kavetsky Institute of Experimental Pathology, Oncology and Radiology of the National Academy of Sciences of Ukraine and the German Collection of Microorganisms and Cell Cultures of the Leibniz Institute (DSMZ) (Germany). 

Adherent (monolayer) cells were cultured in DMEM (high-glucose Dulbecco’s modified Eagle’s medium, Sigma, Ronkonkoma, NY, USA) in the presence of 10% fetal calf serum (FCS), 2 mM L-glutamine, 50 IU/mL penicillin, and 50 μg/mL streptomycin in a humidified atmosphere with 5% CO_2_ at 37 °C. The cells were reseeded every 72 h by diluting the cells in the culture medium at a ratio of 1:5 [37]. Trypsin treatment (1:10 in phosphate-buffered saline (PBS)) was used for substrate-dependent cells. 

The number of live/dead cells was counted in a Goryaev chamber using an Olympus “SKH 41SF” light microscope (Tokyo, Japan) and using a 0.4% solution of the vital dye trypan blue, which stains dead cells with a damaged membrane in blue while living cells remain unstained [38].

### 3.7. Assessment of Cell Viability Using the MTT Test

Cell viability was assessed using the recovery rate of MTT (3-[4, 5-dimethylthiazol-2-yl]-2,5-diphenyl tetrazolium bromide). The method is based on the ability of mitochondrial respiratory chain dehydrogenases to convert MTT to formazan [39]. The MTT test was performed in 96-well plates (GreinerBioOne, Frickenhausen, Germany) at a concentration of cells in a well of (5–10) × 10^3^ per 100 μL. An amount of 10 μL of MTT solution (4 mg/mL phosphate buffer solution) was added to each well and incubated for 2 h at 37 °C, after which the tablets were centrifuged (600× *g*, 7 min) and the supernatant was collected. The supernatant was stored at 4 °C in the dark for 20 h. One hundred microliters of a concentrated solution of dimethylsulfoxide was added to the resulting formazan precipitate. After 15 min, extinction was measured on a spectrophotometer (μQuant, BioTEK, Winooski, VT, USA) at λ = 570 nm. Cell viability was expressed as a percentage of the control. 

Data processing and calculation of the semi-inhibitory concentration (IC_50_) values were performed using the specialized software GraphPad Prism 7 (GraphPad Software Inc., Boston, MA, USA). Individual concentration–dependence curves were constructed by determining the logarithm of the concentrations of the studied compounds compared to the corresponding normalized percentage of cell viability values using nonlinear regression. 

### 3.8. Assessment of ROS Production in Cells 

Intracellular production of reactive oxygen species (ROS) was assessed using the probe 2,7-dichlorodihydrofluorescein diacetate DCFH-DA (Sigma-Aldrich Co., St. Louis, MO, USA), which was added to the cell incubation medium (2 × 10^6^/mL) until the final 5 µM concentration in the sample was reached [40]. The fluorescence intensity of the probe was recorded for 45 min on a Tecan Infinite M200 Pro microplate reader (Männedorf, Switzerland). 

### 3.9. Assessment of Caspase 3/7 Activity in Cells 

Caspase 3/7 activity was determined using the Promega Caspase-Glo^®^ 3/7 Activity assay kit (Madison, WI, USA) in accordance with the manufacturer’s protocol. After 24 h of incubation with the studied compounds, the cells (3 × 10^3^) were kept for 10 min at room temperature. An equal volume of Caspase-Glo 3/7 reagent was added and the mixture was mixed gently on a plate shaker (300 rpm) for 1 min. The samples were incubated at room temperature for 1 h. The luminescence of the samples was recorded on a microplate reader Tecan Infinite M200 Pro (Männedorf, Switzerland).

It should be noted that the copolymer–drug nanocomplex was not washed at any stage of the studies. 

### 3.10. Statistic 

The statistical processing of the research results was carried out using the generally accepted methods of variation statistics, particularly the *t*-test and one-/two-way ANOVA. The difference in the values of *p* ≤ 0.05 was considered statistically significant. The experiments were conducted in at least five parallels in each variant. Microsoft Excel 2010 and GraphPad Prism 7 were used for graphing and data processing.

## 4. Conclusions

These results allow us to conclude that the star-like D-g-PNIPAM copolymers can be effective nanocarriers for Cis-Pt and Dox chemotherapeutic agents. It was proved that the specific molecular structure of the copolymers affects the in vitro antitumor efficacy of the synthesized nanocomplexes. More pronounced cytotoxic effects were observed under the action of the D6-g-PNIPAM-Cis-Pt and D6-g-PNIPAM-Dox nanocomplexes (the D6-g-PNIPAM copolymer has a more rigid structure and, therefore, a lower ability to produce conformational changes; as a result, it has a lower tendency to promote aggregation processes) on sensitive and resistant LLC cells. This was evidenced by a decrease in cell viability, an increase in intracellular ROS levels, and a decrease in ATP content and caspase 3/7 activation compared to the effect of free chemotherapeutic agents at an equivalent concentration. This requires further testing of in vivo models for possible use in cancer therapy.

## Figures and Tables

**Table 1:** Molecular parameters of synthesized D-g-PNIPAM copolymers.

Polymer	M_v_ × 10^−6^, g/mol	M_n_ × 10^−6^, g/mol	M_v_/M_n_	N, % Dextran Component
D6-g-PNIPAM	0.674	0.453	1.49	0.9
D70-g-PNIPAM	1.030	0.674	1.53	6.8

**Table 2:** IC_50_ values under the influence of the studied compounds for sensitive and resistant LLC cells.

IC_50_, μM by Chemotherapeutic Agent	LLC Cells		LLC/DoxR Cells	
	24 h	48 h	24 h	48 h
Cis-Pt	45.86	40.30	52.82	78.98
D6-g-PNIPAM-Cis-Pt	31.32	12.44	16.49	13.95
D70-g-PNIPAM-Cis-Pt	38.40	16.06	22.72	19.17
Dox	2.89	4.33	10.38	11.90
D6-g-PNIPAM-Dox	1.92	2.36	6.67	5.14
D70-g-PNIPAM-Dox	3.09	3.12	8.18	7.31

## Data Availability

Data are contained within the article.
